# First detection and autochthonous transmission of monkeypox virus clade Ib in the Netherlands, October to November, 2025

**DOI:** 10.2807/1560-7917.ES.2026.31.3.2500958

**Published:** 2026-01-22

**Authors:** Jelte Elsinga, Celine van de Ven, Anne de Vries, Hester Coppoolse, Mariska Petrignani, Brigitte van Cleef, Riemer van Markus, Nora Carpay, Catharina E van Ewijk, Sjoerd Rebers, Aldert Bart, Karin J von Eije, Brenda Westerhuis, Sylvia Bruisten, Leonard Schuele, Marjan Boter, Richard Molenkamp, Bregtje Lemkes, Suzanne Geerlings, Henry JC de Vries, Marion Koopmans, Marcel Jonges, Bas B Oude Munnink, Matthijs RA Welkers

**Affiliations:** 1 Amsterdam UMC location AMC, University of Amsterdam, Department of Medical Microbiology and Infection Prevention, Amsterdam, the Netherlands; 2 Amsterdam Institute for Immunology and Infectious Diseases (AII), Infectious Diseases, Amsterdam, the Netherlands; 3 Department of Infectious Diseases, Public Health Service Haaglanden, Den Haag, the Netherlands; 4 Department of Infectious Diseases, Public Health Service Gooi and Vechtstreek, Bussum, the Netherlands; 5 Department of Infectious Diseases, Public Health Service Amsterdam, Amsterdam, the Netherlands; 6 Amsterdam UMC location AMC, University of Amsterdam, Department of Internal Medicine, Amsterdam, the Netherlands; 7 Centre for Infectious Disease Control, National Institute for Public Health and the Environment (RIVM), Bilthoven, the Netherlands; 8 Tergooi MC, Department of Medical Microbiology, Hilversum, the Netherlands; 9 Department of Viroscience, Erasmus University Medical Center, Rotterdam, the Netherlands; 10 Amsterdam UMC location AMC, University of Amsterdam, Department of Dermatology, Amsterdam, the Netherlands; 11 Centre for Sexual Health, Department of Infectious Diseases, Public Health Service of Amsterdam, Amsterdam, the Netherlands; 12 Amsterdam Institute for Global Health and Development, Amsterdam, the Netherlands

**Keywords:** mpox, MPXV, outbreak, Netherlands, clade Ib, whole genome sequencing, epidemiology

## Abstract

In October–November 2025, eight autochthonous cases of monkeypox (MPXV) clade Ib virus infection were reported in the Netherlands. All cases were men who have sex with men aged 25–65; none required hospital admission or antiviral treatment. Phylogenetic analysis combined with contact tracing suggest multiple introductions or cryptic circulation with onwards transmission within the community. Highly related international sequences were identified dating back to August 2025, indicating sustained global community transmission of clade Ib outside the African continent.

In October 2025, the first case of autochthonous mpox caused by infection with monkeypox virus (MPXV) clade Ib was diagnosed in the Netherlands. Following this initial case, seven additional cases were diagnosed by late November. Here, we describe the clinical presentation, transmission dynamics, public health response and phylogenetic analysis.

## Clinical presentation

In early October 2025, a man who has sex with men (MSM) living with HIV, aged 35–40 years, presented with malaise, abdominal pain, rectal tenesmus and mucous anorectal discharge in the emergency department at a hospital in Amsterdam. Physical examination showed 4–5 small pustular peri-anal lesions, without other skin lesions. Based on the similarities to mpox clade IIb clinical presentations in the Netherlands since 2022, mpox infection was suspected [1]. Rectal swabs were PCR-positive for MPXV clade Ib. 

In the following 6 weeks, seven more clade Ib mpox cases were diagnosed in the Netherlands, and later confirmed with whole genome sequencing. The main clinical findings for all eight cases are summarised in the Table. No indication for a major difference in clinical presentation of clade Ib infections was noted compared to clade IIb infections. None of the eight cases required hospitalisation or antiviral treatment. None of them had received a recent (< 2 years) vaccination for mpox. Concomitant bacterial sexually transmitted infections (STIs) were identified in three of eight cases (chlamydia, gonorrhoea, syphilis) and, in one case, a gastrointestinal infection (*Campylobacter* spp.).

**Table:** Overview of symptom onset after exposure, age group, HIV status, initial clinical presentation and most likely source of infection of mpox clade Ib cases, the Netherlands, October–November 2025 (n = 8)

Case	Symptom onset in days after exposure^a^	Age group in years	HIV status	Main clinical picture^b^	Most likely source^a^	Co-infection	Identified high-risk contacts through contact tracing^c^
1	4	35–40	Positive	Perianal pustules followed by generalised rash (arms/legs), general malaise	Condomless anal receptive sexual contact with one male person early Oct 2025 in a private residence	Anal chlamydia; *Campylobacter* gastroenteritis	1 (household contact)
2	4	30–35	Negative	Perianal pain, papules, general malaise and headache	Sexual contact with one male person in Venue A mid-Oct 2025	None detected	3 (sex partner Case 6, and 2 household contacts)
3	6	25–30	Negative	Pustules on palm, hand, axilla, heel, general malaise, sore throat	Intimate skin contact with multiple male persons in Venue A mid-Oct 2025	None detected	1 (partner)
4	8	30–35	Positive	Hand, facial and thoracic lesions, anal pain, general malaise	Condomless anal insertive and receptive sexual contact with multiple male persons in Venue A mid-Oct 2025	Anal gonorrhoea	None
5	10	60–65	Negative	Chest/leg pustules, penile ulcers, followed by generalised rash	Condomless insertive anal sexual contact with three male persons in Venue A mid-Oct 2025	None detected	None
6	8	40–45	Negative	Papules on arms, mouth, chest with fever and general malaise	Oral and anal receptive sexual contact with Case 2 end-Oct in a private residence	None detected	None
7	9	55–60	Negative	Scrotal pustules followed by generalised rash, general malaise	Sexual contact with two male persons at Venue A end-Oct	None detected	None
8	34	40–45	Positive	Generalised rash and pustules, abdominal pain, general malaise	Kissing with a male person in a bar mid-Oct 2025	Syphilis	1 (partner)

^a^ Most likely transmission event as judged by the public health services based on source and contact tracing; the following information is unknown if not further specified: receptive or insertive, with condom or condomless, oral or/and anal sexual contact.

^b^ All cases presented with a prodrome of malaise/fatigue, none required hospitalisation, all recovered while self-isolating at home.

^c^ High-risk contacts identified during contact tracing according to the definition in the Dutch guidelines [4]. Contacts are defined as either low-risk or high-risk. High-risk contacts include e.g. contact with lesions, kissing, but could also include living in the same household without intimate contact (household contacts).

## Transmission characteristics

Four (Cases 2–5) of the eight cases reported sexual contacts at the same sex venue (Venue A) on a night in mid-October in Amsterdam. This sex venue is a large space that can accommodate around 1,000 visitors per weekend. In addition, a fifth case (Case 7) visited Venue A, but at a later point in time (end-October). Case 6 had a direct epidemiological link with Case 2; they had sex in a private residence. For Case 1, the most likely transmission setting was in a private residence. Case 8 self-reported one high-risk moment (kissing) at a bar in Amsterdam on mid-October but symptoms only appeared 34 days later. This is longer than the 21 days incubation period as defined by the Dutch guidelines [2]. However, it was the only risk moment identified through source tracing, and a recent study estimated that 5% of mpox clade Ib infections might have an incubation period longer than 32.3 days [3]. 

## Public health response

Directly after initial reporting to regional public health services, each case was contacted and advised self-isolation combined with hygiene measures until fever resided for at least 3 days and the remaining skins lesions could be covered by clothing or bandages. Contact tracing was performed and contacts were identified according to the definition in the Dutch guidelines [4]. All identified high- and low-risk contacts were informed and monitored for the development of symptoms up to 21 days after their last exposure [2]. Post-exposure vaccination (MVA-BN; Imvanex) was offered to at least one previously unvaccinated high-risk contacts who did not accept the vaccine.

Since all Venue A visitors were anonymous, public health authorities could not retrospectively alert other visitors. To prevent future transmission, mpox-related information was published on the homepage of venue’s website, and engaging vaccination campaigns were organised on site, with subsequent campaigns scheduled for the remainder of 2025. To further improve mpox awareness in the key populations, mpox counselling was integrated into existing hepatitis B consultations at the public health services. In MSM with potential clade Ib infections, similar to the clade IIb presentations, bacterial STIs need to be excluded and prophylactic interventions offered, such as condom use, pre-exposure prophylaxis against HIV and hepatitis B vaccination. In addition, general practitioners, microbiological laboratories, dermatologists and infectious disease clinicians nationwide received an update to ensure clinical awareness. Different non-profit organisations, including STI prevention and community support organisations, distributed educational posters and messages to sex venues. Additionally, online information materials engaging key populations on multiple official websites were updated to include mpox guidance.

## Phylogenetic analysis

PCR-positive samples from the eight mpox cases were subjected to whole genome sequencing using a previously published or an adapted primer scheme (see Supplementary Materials for details). Genome coverages varied between 95.6% and 99.8%, reaching near full genome coverage for all eight cases. Phylogenetic analysis revealed high sequence similarity of all eight samples with a maximum difference of eight nucleotides between cases (see the Figure). The sequences of two epidemiologically linked cases (2 and 6) were identical, whereas other sequences had up to eight nucleotide differences, suggesting more widely dispersed virus circulation. For Case 8, a high-risk moment in a bar was identified as the most likely source. However, the obtained sequence was identical to Cases 2 and 6, suggestive for a link to the initial infections at Venue A or from a shared unknown third source.

**Figure:**
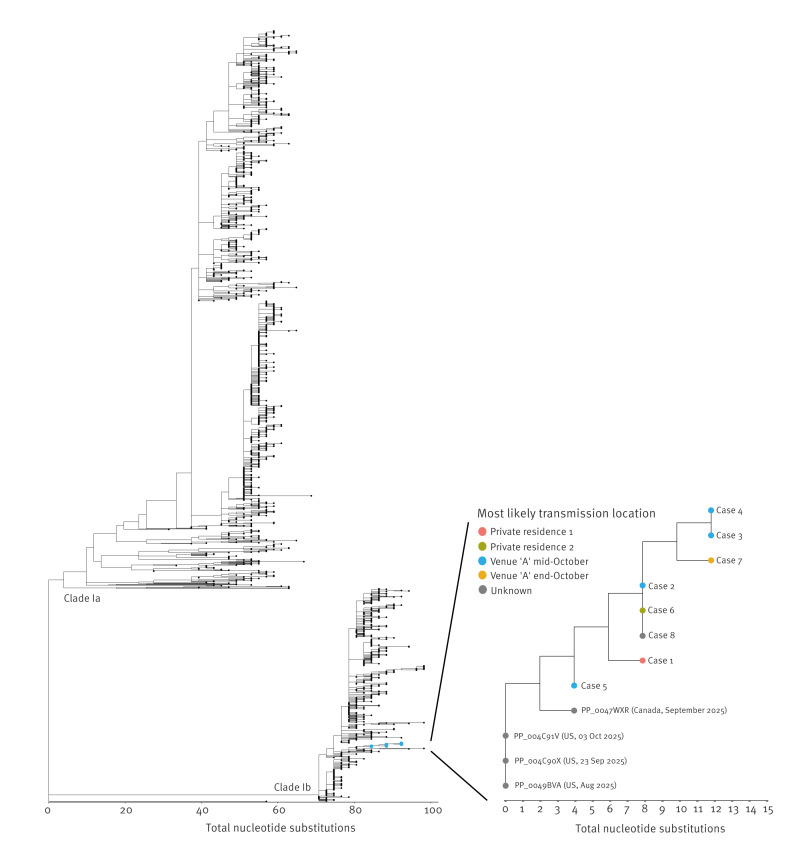
Phylogenetic tree containing sequences of the Dutch MPXV clade Ib infections, October–November 2025 (n = 8), within the international context of all available clade Ib sequences on Pathoplexus, 13 October 2023–10 October 2025 (n = 487)

US: United States.

Phylogenetic tree (left side) containing available MPXV clade I viral sequences with the sequences highlighted in blue that were generated as part of this study. Phylogenetic tree (right side) with a zoom of the branch containing the clade Ib sequences generated as part of this study with the colours corresponding to the most likely transmission route. To provide context, a background set of sequences from Pathoplexus was used, which was made available via the Nextstrain website (see Supplementary Materials for details). Sequences were extracted on 6 December 2025, with sampling dates from 13 October 2023 to 10 October 2025.

## Discussion

Multiple cases of MPXV clade Ib infection could be linked to a specific night at Venue A. However, Case 1 was symptomatic 4 days prior to that specific night and had no epidemiological link with Venue A. In addition, the viral sequence had multiple mutations compared to the cases that visited Venue A. This suggests either previously undetected circulation, or at least two possible introductions with MPXV clade Ib, which is likely given the recent rise of mpox clade Ib cases in Europe, United States and Canada among travellers and MSM [5,6]. Interestingly, the genetically most similar international sequences were sampled in Canada and the United States, dating back to August 2025, clearly indicating global circulation and spread of MPXV clade Ib in a highly connected international MSM network (see the Figure).

Four cases visited Venue A on the same date, while one case visited the same venue 2 weeks later. Considering the international audience at the large sex venue, it makes wider circulation of closely related clade Ib strains at the same place possible. While the venue is considered a high-risk transmission setting, the genomic diversity suggests that the virus already was more widespread in this community. This is underlined by the identical strains of Case 2, Case 6 and Case 8, while evidence for direct transmission could not be identified. Furthermore, it is yet unclear what the normal genetic variation of a clade Ib virus is within a person after several days of infection. From previously investigated clade IIb infections in the Netherlands, the viral evolutionary rate was significantly higher (11–12 mutations per year) compared to the estimated 1–2 nucleotide changes per year, mostly due to APOBEC-3 related mutations [7]. Also, in 2022, we investigated the moment of introduction of the clade IIb strain in the Netherlands and found likely introduction a few weeks before the first identified cases with very limited viral evolution within that timeframe [8]. To gain more insight in MPVX genomic variability, within-host evolution, as well as occurrence of potential recombinant variants or deletions variants with several clades of MPXV currently co-circulating in the geographical region, it is imperative to have accurate genomic surveillance via whole genome sequencing available [7], as recently demonstrated in the United Kingdom [9]. This should also include improved data sharing and global surveillance as it facilitates the understanding of transmission routes/risk factors in support of effective development of mitigation strategies. 

Between November 2025 and 15 January 2026, four additional clade Ib cases have been reported in the Netherlands, indicating continued transmission [10]. This study was limited by the data collection, since it was based on data from clinical files and therefore there were missing data on clinical presentation. Also, combined with the low number of cases, it does not allow for a valid comparison concerning clinical presentation and transmissibility to MPXV clade IIb strains. 

## Conclusion

With the introduction and subsequent autochthonous transmission of MPXV clade Ib viruses in the Netherlands, as well as other European countries, there is now co-circulation of multiple MPXV clades within Europe. Clinical awareness in unvaccinated high-risk groups, combined with early diagnostic testing via PCR, is of key importance to allow for timely public health response. Rapid data sharing of genome sequences via public databases is essential for monitoring both the national and international spread of MPXV clade Ib. Continued attention should be given to improve mpox awareness in key populations as well as the availability of mpox vaccination. Further research is needed to assess potential differences in intrinsic transmissibility or clinical characteristics between the different clades of MPXV.

## Data Availability

The MPXV clade Ib sequences generated in this study have been made available via GISAID with ID numbers EPI_ISL_20233004.2 (Case 1), EPI_ISL_20233003.2 (Case 2), EPI_ISL_20260546 (Case 3), EPI_ISL_20238028 (Case 4), EPI_ISL_20238029 (Case 5), EPI_ISL_20260547 (Case 6), EPI_ISL_20260548 (Case 7) and EPI_ISL_20275106 (Case 8).

## Supplementary Data

183000 Supplement
