# Addendum for Euro Surveill. 2026;31(3)

**DOI:** 10.2807/1560-7917.ES.2026.31.8.260226a

**Published:** 2026-02-26

**Authors:** Eurosurveillance editorial team

**Affiliations:** 1 European Centre for Disease Prevention and Control (ECDC), Stockholm, Sweden

The article ‘First detection and autochthonous transmission of monkeypox virus clade Ib in the Netherlands, October to November, 2025’ by Elsinga et al., published on 22 January 2026, includes usage of sequence data with restricted use from the platform Pathoplexus as a background set. At publication, this dataset was not referenced in the Data availability statements.

At the request of the corresponding author, this oversight was rectified on 24 February 2026, and the data availability statement was updated to include the reference to the used Pathoplexus background dataset. The authors apologise for the initial omission of this reference. 

