# Supplementary material for: First detection and autochthonous transmission of monkeypox virus clade Ib in the Netherlands, October to November, 2025
Source: Euro Surveill. 2026 Jan 22;31(3):2500958. doi: 10.2807/1560-7917.ES.2026.31.3.2500958 (PMC12848989; doi:10.2807/1560-7917.ES.2026.31.3.2500958)
Supplement: Supplement [file 25-00958_WELKERS_Supplement.pdf]

This supplementary material is hosted by Eurosurveillance as supporting information alongside the article "First detection and autochthonous transmission of monkeypox virus clade Ib in the Netherlands, October 2025" on behalf of the authors who remain responsible for the accuracy and appropriateness of the content. The same standards for ethics, copyright, attributions and permissions as for the article apply. Eurosurveillance is not responsible for the maintenance of any links or email addresses provided therein.

Mpox virus molecular diagnostics and clade typing PCR

Moleculair diagnostic procedures differed slightly depending on the performing laboratory. For AmsterdamUMC, in total 200µl of clinical sample was used for DNA extraction using the automated Roche MagNAPure platform (Roche Diagnostics International, Rotkreuz, Switzerland) and eluted in a total volume of 50µl. Molecular mpox virus diagnostics was performed using two (F3L and G2R) mpox virus aspecific targets in a duplex real-time PCR assay with internal amplification controls. Both F3L and G2R primer and probe sequences were obtained from literature, but the assays were in-house modified and validated to run on FLOW lab automation (Roche Diagnostics International, Rotkreuz, Switzerland) [1,2]. For ErasmusMC, in total, 400uL of clinical sample was used for DNA extraction using the external lysis protocol on the automated Roche MagNAPure platform (Roche Diagnostics International, Rotkreuz, Switzerland) and eluted in a total volume of 100uL. Molecular diagnostics was performed using a generic orthopox PCR [12] combined with a mpox specific PCR [2].
For clade typing PCR's in both centres, the assays recommended by the United States Centers for Disease Control and Prevention (CDC) for clade I (C3L) and clade II (G2R_WA) were combined with the recently published Schuele et al. typing PCR to detect the novel clade Ib variant [3].

Mpox virus whole genome sequencing

Whole genome amplification at the AmsterdamUMC was performed using two different primer protocols. First, we used our previously published protocol, which is described in detail on https://www.protocols.io/private/974ACB78F83911EC998C0A58A9FEAC02. However, we noticed that for clade Ib samples this led to multiple amplicon dropouts and reduced genome coverage to 80-85%. We then aimed to develop a new primer set capable of amplifying both mpox virus clade Ib and IIb samples equally well. A representative set of sequences for clade Ib were downloaded from GenBank and for clade IIb sequences we used previously generated in-house sequences from Dutch samples. Primers were then designed using PrimalScheme3 resulting in a total of 194 primers [4]. To reduce PCR inhibition, we split the amplification into five separate PCR reactions (pool 1 till 5) with 54, 48, 48 and 44 primers per pool, respectively. Pool 5 consisted of 8 primer pairs that were also present in pools 1 to 4, but repeatedly had low read number in multiple samples. DNA amplification was performed as follows: per reaction 10 µl Q5 hotstart enzyme (New England Biolabs), 6 µl of 10µM primer pool working solution and 4 µl of DNA eluate. In the PCR, settings were as follows: denaturation of 2' for 98°C followed by 40 cycles of 15 seconds at 98°C, 5 minutes at 64°C followed by a final cooldown to 10 °C. After the multiplex PCR, purification was performed using the AMPure XP beads (Beckman Coulter). Library quantification was performed by using Qubit dsDNA HS assays kits (Thermo Fisher). After normalization each individual sample was barcoded with the Oxford Nanopore SQK-MAB114-24 Microbial Amplicon Barcoding Sequencing for 16S and ITS (Oxford Nanopore Technologies, ONT) or separately the rapid barcoding kit SQK-RBK114-96 (ONT). Oxford Nanopore sequencing was performed using Nanopore Minion R10.4 flowcells on a Nanopore Gridion (ONT).
Whole genome sequencing at the ErasmusMC was performed using the artic-inrb-mpox v1.0.0 scheme (https://github.com/quick-lab/primerschemes/tree/main/primerschemes/artic-inrb-mpox/2500/v1.0.0).

DNA amplification was performed as follows: in a total volume of 25 µl 0.33 µl Q5 enzyme, 0.75 µl $MgCl_2$, 1 µl of 20 µM primers and 5 µl of eluted DNA. In the PCR, settings were as follows: denaturation of 45" for 98°C followed by 35 cycles of 15 seconds at 98°C, 5 minutes at 65°C followed by a final cooldown to 12 °C.
Sequencing libraries were prepared using the SQK-NBD114.24 Native Barcoding Kit v14 24 (ONT) [12].

Sequencing data analysis

Resulting pod5 files were merged into a single pod5 file and either rebasecalled using Dorado v.1.3.0

(Oxford Nanopore Technologies, ONT) or the generated fastq data was used from the live basecalling performed during sequencing using MinKNOW software version 24.11.8 (ONT) with the super-accurate basecalling model. Using the obtained fastq files an initial quality control step was performed using fastp version v.0.23.4 [5] using the following settings: -A -l 50 --length_limit 3500 -q 10 -5 cut_front_window_size=5 cut_front_mean_quality=10 -3 cut_tail_window_size=5 cut_tail_mean_quality=10. Reads passing quality control were subsequently mapped to the human genome GRCh38 to remove contaminant human reads using minimap2 version 2.18-r1015 with default settings [6]. Primers were removed from the reads that did not map to the human genome using the AmpliGone package version v.2.0.2 using settings "--amplicon-type end-to-mid" and a fasta file containing the primer sequences as input [7]. Resulting reads were then remapped using minimap2 version 2.18-r1015 against clade Ib sequence hMpxV/Japan/KIH-G250014/2025|EPI_ISL_20193695|2025-09-12 and the resulting sam-file was converted to bam-file format, sorted and indexed using samtools v.1.104 [8]. A consensus sequence was generated using the TrueConsense package (https://github.com/RIVM-bioinformatics/Trueconsense) using settings (-cov 30 -noambig) and additionally a coverage overview per position from the sorted BAM file was generated using pysamstats v.1.1.2 (https://github.com/alimanfoo/pysamstats) with setting -t variation_strand -D 10000000. Resulting consensus sequences were manually checked in case of any discrepancies and finally deposited online in GISAID.

Phylogenetic analysis

For phylogenetic analysis the latest available Nextstrain mpox workflow (available at https://github.com/nextstrain/mpox) was downloaded at December 6th 2025 [9]. The clade-i config file was changed to include the locally generated sequences. The included Pathoplexus dataset, that was automatically downloaded from data.nextstrain.org, included restricted sequences. Subsampling was done with using the command "--query 'clade in ["Ib"] | input_name == "local"' to always included locally generated sequences and available clade Ib sequences from the Pathoplexus dataset. No changes were made to the masking settings. The resulting tree was combined with generated metadata using the ggtree package in Rstudio v.2025.09.2 build 418. with R version 4.5.2 [10, 11].

References

1. Maksyutov RA, Gavrilova E V., Shchelkunov SN. Species-specific differentiation of variola, monkeypox, and varicella-zoster viruses by multiplex real-time PCR assay. J Virol Methods [Internet] 2016;236:215-20. Available from: http://dx.doi.org/10.1016/j.jviromet.2016.07.024

2. Li Y, Zhao H, Wilkins K, Hughes C, Damon IK. Real-time PCR assays for the specific detection of monkeypox virus West African and Congo Basin strain DNA. J Virol Methods. 2010;169(1):223-7.

3. Schuele Leonard, Masirika Leandre Murhula, Udahemuka Jean Claude, Siangoli Freddy Belesi, Mbiribindi Justin Bengehya, Ndishimye Pacifique, Aarestrup Frank, M, Koopmans Marion, Oude Munnink Bas B, Molenkamp Richard, GREATLIFE MPOX group. Real-time PCR assay to detect the novel Clade Ib monkeypox virus, September 2023 to May 2024. Euro Surveill. 2024;29(32):pii=2400486

4. Chris Kent, Andrew D Smith, John Tyson, Dominika Stepniak, Eddy Kinganda-Lusamaki, Tracy Lee, Mia Weaver, Natalie Sparks, Thomas Brier, Lauren Landsdowne, Sam Wilkinson, Rachel Colquhoun, Áine O'Toole, Placide Mbala-Kingebeni, Ian Goodfellow, Andrew Rambaut, Nicholas Loman, Joshua Quick
bioRxiv 2024.12.20.629611; doi: https://doi.org/10.1101/2024.12.20.629611

5. Hifu Chen. 2025. fastp 1.0: An ultra-fast all-round tool for FASTQ data quality control and preprocessing. iMeta 2025: https://doi.org/10.1002/imt2.107

6. Li H. Minimap2: Pairwise alignment for nucleotide sequences. Bioinformatics 2018;34(18):3094–100.

7. Zwagemaker, F., Hajji, K., Raaijmakers, G., Schmitz, D., Kroneman, A. AmpliGone [Computer software]. https://doi.org/10.5281/zenodo.7684307

8. Li H, Handsaker B, Wysoker A, Fennell T, Ruan J, Homer N, Marth G, Abecasis G, Durbin R, and 1000 Genome Project Data Processing Subgroup, The Sequence alignment/map (SAM) format and SAMtools, *Bioinformatics* (2009) 25(16) 2078-9

9. James Hadfield, Colin Megill, Sidney M Bell, John Huddleston, Barney Potter, Charlton Callender, Pavel Sagulenko, Trevor Bedford, Richard A Neher, Nextstrain: real-time tracking of pathogen evolution, *Bioinformatics*, Volume 34, Issue 23, December 2018, Pages 4121–4123, https://doi.org/10.1093/bioinformatics/bty407

10. R Development Core Team. R: A language and environment for statistical computing [Internet]. 2008;Available from: http://www.r-project.org

11. Yu G, Smith DK, Zhu H, Guan Y, Lam TTY. Ggtree: an R Package for Visualization and Annotation of Phylogenetic Trees With Their Covariates and Other Associated Data. Methods Ecol Evol 2017;8(1):28–36.

12. Quick, Josh, and Dominika Stepniak. "ARTIC LoCost Amplicon Sequencing Protocol (SQK-NBD114)." (2025). https://dx.doi.org/10.17504/protocols.io.5jyl885p7l2w/v1

Supplementary table S2. Primers and sequences for AmsterdamUMC mpox clade I/II whole genome sequencing protocol

**Primer name**	**Sequence (5' -> 3')**	**Tm**
Pool1_mPox_AUMC_1_LEFT	CGTAGGAAACTCTAGAGGGTAAGAAAAATC	65
Pool1_mPox_AUMC_1_RIGHT	CACCGTCTCTTCCACAGATAAATGC	66
Pool1_mPox_AUMC_13_LEFT	GTGTCTGTTTATACAATCAATTATACTAGGATCCA	64
Pool1_mPox_AUMC_13_RIGHT	GGTTTACAAATTAGTCTTACTCTTTTGTATCGC	64
Pool1_mPox_AUMC_17_LEFT	CTTCGTCTATTACACCGCCTCCT	67
Pool1_mPox_AUMC_17_RIGHT	GAATCTTCTTTTCCGGATCATTCGCT	66
Pool1_mPox_AUMC_23_LEFT	TGTTTGCTGCTAATCGCGACAA	66
Pool1_mPox_AUMC_23_RIGHT	ATAAGAAAACCAACAGAGTATTATGCTTTGAC	64
Pool1_mPox_AUMC_27_LEFT	AGGCAATGTAACGCGTCTGTTTT	66
Pool1_mPox_AUMC_27_RIGHT	TCATTGTGAACCTAGACTACCGAACC	66
Pool1_mPox_AUMC_31_LEFT	TTGCCTTGGCTAATTGTCTTTCTCTT	65
Pool1_mPox_AUMC_31_RIGHT	GGGGGAATAGGTCTATCAATTAGTAATATTCGT	65
Pool1_mPox_AUMC_33_LEFT	AGTAAACAGTCCATCGTTGCAATAATAAAAG	64
Pool1_mPox_AUMC_33_RIGHT	TTTGGTTGTTTGTTTATTAATTCTTCCGGT	64
Pool1_mPox_AUMC_35_LEFT	GTCAAGGATATTAATATCGACTTACTATCGTCAT	64
Pool1_mPox_AUMC_35_RIGHT	AGTAGAAGAATTAAAATCCGAGTATGATATACTCC	63
Pool1_mPox_AUMC_38_LEFT	TGGAACCGCACCCAATTGCT	69
Pool1_mPox_AUMC_38_RIGHT	CCGGCTCTATAGAACAAATCTACCACAG	67
Pool1_mPox_AUMC_42_LEFT	CGATCCTAAAGGTAAATGTAAATTCAGCGG	66
Pool1_mPox_AUMC_42_RIGHT	CGTTAATAGGTTCTGACTTTTTATGAAGAGC	64
Pool1_mPox_AUMC_46_LEFT	GTAGTATCTAAAACTACGTTCGATGAATTCCT	64
Pool1_mPox_AUMC_46_RIGHT	TCCGATAGTTTATCCTCGTTAAAGACATCT	65
Pool1_mPox_AUMC_5_LEFT	ACTCCTATAGTTTCCACATCAACCAAGT	66
Pool1_mPox_AUMC_5_RIGHT	TCAAAGAAGATGAAACCGTTCGTACC	65

Pool1_mPox_AUMC_52_LEFT	CCAAACATACATCTATAAATTTGACGCTTTTCT	64
Pool1_mPox_AUMC_52_RIGHT	GGCCAATGTATCTAGGGGGAAAGATA	66
Pool1_mPox_AUMC_55_LEFT	ACAAATCTTGTGAACTAGACATATCGGTAAA	64
Pool1_mPox_AUMC_55_RIGHT	AAGACAATAATCTCTGAAGAATATCTATCGAAGG	64
Pool1_mPox_AUMC_57_LEFT	ATCCTAGACCAGTAAACAGTTTCCACT	65
Pool1_mPox_AUMC_57_RIGHT	TTGTGAACAAAAGCTACGACCAAATTC	64
Pool1_mPox_AUMC_61_LEFT	AAACTACTACAATGATCTATCGACATACCATC	64
Pool1_mPox_AUMC_61_RIGHT	ACTTTCTTGTTTAAATCCGAGTTTATCTACAGA	64
Pool1_mPox_AUMC_63_LEFT	TGTCACAATATGAATACACTACAGTTCTTCTAG	64
Pool1_mPox_AUMC_63_RIGHT	GAGACAGCAAAACAATATTGACCGTTATAAAT	64
Pool1_mPox_AUMC_67_LEFT	AAGAGGAAATATTCAGTATATAGATATGCCTTAGC	64
Pool1_mPox_AUMC_67_RIGHT	TGATATCTCTGGCTAAAAAGATTGATGTACAG	64
Pool1_mPox_AUMC_69_LEFT	ACTTTGTCCGTAAAACATAAATATAAATACCCG	63
Pool1_mPox_AUMC_69_RIGHT	GTCTAGTATCAGGTCTAGGCAATCTAGC	66
Pool1_mPox_AUMC_73_LEFT	CGTCTACATTGATAGTGATCATATACGTGTT	64
Pool1_mPox_AUMC_73_RIGHT	TCAATGTCTCCATACTGTTTAATAACCAAATCA	64
Pool1_mPox_AUMC_75_LEFT	AGATGTTAGTACAGTGTTATAAATGGATGAAGC	65
Pool1_mPox_AUMC_75_RIGHT	GTCAAGTTTACTAAATCGTTTAGATGAGTAGATTC	63
Pool1_mPox_AUMC_77_LEFT	CCATGGATGGTGTTATCGTATACTGTCT	66
Pool1_mPox_AUMC_77_RIGHT	AAACTTAAACCACCATCAAAAATCCATGTT	64
Pool1_mPox_AUMC_78_LEFT	TCTGATGATGTTCAAACTAATACATCTGAAACA	64
Pool1_mPox_AUMC_78_RIGHT	ACATTAATGGATTTGTATAAACCAAATGCCTTG	64
Pool1_mPox_AUMC_89_LEFT	TGATCAACGATCGAATAACAATGTAAGTACTA	64
Pool1_mPox_AUMC_89_RIGHT	CGTATCTATTATACCAGTTATAGCCGTTATAGC	64
Pool1_mPox_AUMC_9_LEFT	TCTCTGTATTCTACGATAGAGTGCTATTTTAAGA	64
Pool1_mPox_AUMC_9_RIGHT	CAATGGTTTATGTCCGTAGTGATATAAAATGG	64
Pool1_mPox_AUMC_92_LEFT	TTGTATCTGACTCTGATCAATGTAAACCTATG	64
Pool1_mPox_AUMC_92_RIGHT	TGTGACAATTAACAAGATACAAAAGAGATGC	64
Pool1_mPox_AUMC_97_LEFT	CTCTTCCACAGATAAATGCGAACCC	66
Pool1_mPox_AUMC_97_RIGHT	CGTAGGAAACTCTAGAGGGTAAGAAAAATC	65
Pool2_mPox_AUMC_10_LEFT	CTCTCTAATACAATCAGATATATCTATTGTGTCGG	64
Pool2_mPox_AUMC_10_RIGHT	CGTCGATGTCAGAAATAATCAACAATATTACTG	64
Pool2_mPox_AUMC_14_LEFT	GTACCAGTACCGGTAATCTTGTCGA	66
Pool2_mPox_AUMC_14_RIGHT	GAAAAAGAGATCTGGGTCCAGCATTT	66
Pool2_mPox_AUMC_18_LEFT	GCATTAGGCCACGCTTTTTAAGCC	68
Pool2_mPox_AUMC_18_RIGHT	TGATTATCCACGAATTATATCATATAATCCTCCAC	64
Pool2_mPox_AUMC_2_LEFT	CCACATCGATATAGTTAAATGTATTGCTGGT	65
Pool2_mPox_AUMC_2_RIGHT	ACACTGCGATCTGAATAAACGACTAAAA	65
Pool2_mPox_AUMC_24_LEFT	TCCTGGAATAGAATCTATTTTAATGAGGCTTT	64
Pool2_mPox_AUMC_24_RIGHT	ACGAGCGTTCTGACACAGAGAT	66
Pool2_mPox_AUMC_28_LEFT	GGTTCCTTCTATTGATCTATCGAAAATTGCT	65
Pool2_mPox_AUMC_28_RIGHT	CGGTTATTTCAGTCGTGACTAATCATAAGTTT	65
Pool2_mPox_AUMC_32_LEFT	GTTTGATATACCATTGGTACCGGAGATATAG	64
Pool2_mPox_AUMC_32_RIGHT	AACAAGTAGCTTCAAAGTCTCTAAAACCT	64
Pool2_mPox_AUMC_39_LEFT	ATCCTCTACGGGCTATTGTCTCATG	66

Pool2_mPox_AUMC_39_RIGHT	AGGATCTATGTATCTAACAGGATCTATTGCG	65
Pool2_mPox_AUMC_43_LEFT	CTTAAGAGAGCTACGTTCGGAGATAATAAAG	65
Pool2_mPox_AUMC_43_RIGHT	AGCATATTAATGATCAGAAGTTCGATGATGT	64
Pool2_mPox_AUMC_47_LEFT	TCATAGTCGAACATTTAAGAGATCAAAGCA	64
Pool2_mPox_AUMC_47_RIGHT	CCAACTGGCTACTTCGTTATAAAACTCT	65
Pool2_mPox_AUMC_50_LEFT	TGTATAATTCTCCTGAAAACGATGATGGC	65
Pool2_mPox_AUMC_50_RIGHT	CGTGTCTTTTGATCTTGGAATATCTTTTATTCG	64
Pool2_mPox_AUMC_53_LEFT	CCTTATACAGGTCATTACATACAACTACCAAT	64
Pool2_mPox_AUMC_53_RIGHT	GTAACCAATTAGTATGGGAAAACTTTTTAGCA	64
Pool2_mPox_AUMC_58_LEFT	AAATACATTATCATGCTTGCAATGGCTC	64
Pool2_mPox_AUMC_58_RIGHT	GTGTTAGACAATATATAACAGCACAAGATCAAC	64
Pool2_mPox_AUMC_6_LEFT	TGAAGGACTTTACGTAGAGTGGTGAT	65
Pool2_mPox_AUMC_6_RIGHT	GCTTTACACGCATACCTTTTCAATGAG	65
Pool2_mPox_AUMC_62_LEFT	ATAAAGTACATGATAGGAAATTTCGTGCAAC	64
Pool2_mPox_AUMC_62_RIGHT	AAAATAGATAAATTTGACGTACGGTGACCT	64
Pool2_mPox_AUMC_64_LEFT	ATCACAATTGGAAGCGTCGAATATATCA	64
Pool2_mPox_AUMC_64_RIGHT	ACAGACAGGTCGGTTTTCTCCC	68
Pool2_mPox_AUMC_70_LEFT	GTTACTCTCAACCATTATTAGCGGCAT	65
Pool2_mPox_AUMC_70_RIGHT	CGATGTTACCAATCGTTTGCTGGG	67
Pool2_mPox_AUMC_76_LEFT	CAGATTCTGCCGATTTAGTAGTACTAGGT	66
Pool2_mPox_AUMC_76_RIGHT	TGGTAGATAGACCATCGAATGTATTTTCTAAC	64
Pool2_mPox_AUMC_79_LEFT	AACCATCCGTTATCGAAGAATGGAAAA	65
Pool2_mPox_AUMC_79_RIGHT	CCAATCCGCAAACACAGCACA	67
Pool2_mPox_AUMC_81_LEFT	ACATCATTGTTTCCTTTTAGTGCTCGA	65
Pool2_mPox_AUMC_81_RIGHT	TTGAATAATATGTATTACTATGCCCATGACGA	64
Pool2_mPox_AUMC_84_LEFT	TGGATGAACCATTGTCTCCTGATACC	67
Pool2_mPox_AUMC_84_RIGHT	AGATTTTATTTGCTACACTTATTCTACCGTCT	64
Pool2_mPox_AUMC_86_LEFT	TGTCGTGGACACAACTATTATTTTGATAAAAC	64
Pool2_mPox_AUMC_86_RIGHT	CACGACGTATAGGAAACTCTGACTTATTATT	64
Pool2_mPox_AUMC_88_LEFT	GTTATTAGCGCCGTCCATTTTAAAGC	65
Pool2_mPox_AUMC_88_RIGHT	AATTCTGTTTCATTGACTCCCAATATTTTACTC	64
Pool2_mPox_AUMC_95_LEFT	TGTACGGAAAGAACCATTACAAATATTATCCA	64
Pool2_mPox_AUMC_95_RIGHT	GATCATATCGTATCACATATTGAAACAGAAAGAAG	64
Pool3_mPox_AUMC_11_LEFT	AGTATCCATTTTATATCACTACGGACATAAACC	64
Pool3_mPox_AUMC_11_RIGHT	GCTGCCATAATAATTGATGTTCCGCC	67
Pool3_mPox_AUMC_15_LEFT	ACACACAGTACTATCGACGAAACTTTG	65
Pool3_mPox_AUMC_15_RIGHT	CCAACAAGGTTAGAAAAATAATTCCCAAGATT	64
Pool3_mPox_AUMC_19_LEFT	GTTCACATTGAAACTATGTCAGTAGTTATACATC	63
Pool3_mPox_AUMC_19_RIGHT	TCATCCCCTGAATATCAATGGATGTCT	66
Pool3_mPox_AUMC_21_LEFT	TTTTTATTAAATCAAATGGTCGGCTCTCTG	64
Pool3_mPox_AUMC_21_RIGHT	ATGTGGCCATTTGCATCGGTAC	67
Pool3_mPox_AUMC_25_LEFT	CGCTTCTCCATATTAAGTTGTTTAGTTAGTTG	64
Pool3_mPox_AUMC_25_RIGHT	CGTGTCGTAAAACCGCTTCGTATAC	66
Pool3_mPox_AUMC_29_LEFT	GCAATCGATGTGTCAAAGGTTAAACC	65
Pool3_mPox_AUMC_29_RIGHT	CAATGTTAGGTTCTTTCCTCCTGAAATAATAAAC	64

Pool3_mPox_AUMC_3_LEFT	ACAGATGGATTAAATCGTGTATTCATCGT	64
Pool3_mPox_AUMC_3_RIGHT	GATTTCATCGTCATTACCATAACTAGTTTAAGTC	64
Pool3_mPox_AUMC_34_LEFT	TGGGATTTAAGGTACTAGATGGATCTCC	65
Pool3_mPox_AUMC_34_RIGHT	TGAATTATAGCGACGATTATATGATGAACATCT	64
Pool3_mPox_AUMC_36_LEFT	CCAACATACTTGAATCACCATCCTTTAAAAA	64
Pool3_mPox_AUMC_36_RIGHT	AGAGTGATGGCAAACAACGTTAAGAAA	65
Pool3_mPox_AUMC_40_LEFT	ACTTTGTGATTAGTTTGATGCGATTCAAAA	64
Pool3_mPox_AUMC_40_RIGHT	CGACTGAAATAACAGATCTATCGGCTATCT	66
Pool3_mPox_AUMC_44_LEFT	TGATCTGGATCATCATTGACTATCACAAC	65
Pool3_mPox_AUMC_44_RIGHT	AACACATTAGACATGTATTTACGACAAAAGAAG	64
Pool3_mPox_AUMC_48_LEFT	CACACGCACCATATACTATTACTTATCACG	65
Pool3_mPox_AUMC_48_RIGHT	ATAGCAGACTTTAACAAACGTTTGGTTG	64
Pool3_mPox_AUMC_51_LEFT	GAGGAAGTGATATTTGAAACTCCTAGAGAATT	64
Pool3_mPox_AUMC_51_RIGHT	GGGGATGCTATTGCGGCTGATA	68
Pool3_mPox_AUMC_59_LEFT	CGTTTGGTAATGGCAATGTATTAAGATTAACT	64
Pool3_mPox_AUMC_59_RIGHT	GAAACAGTTAATAAGATTACACCACAGCTTAAA	64
Pool3_mPox_AUMC_65_LEFT	CTTTAATAACAAAGAAAGAGATAGGTCTAACGC	64
Pool3_mPox_AUMC_65_RIGHT	ACAAACTGGAAATTGAATCTCTTAAACGTG	64
Pool3_mPox_AUMC_7_LEFT	TCTAGTAGTAGTTTGACGACCTCAACAT	65
Pool3_mPox_AUMC_7_RIGHT	TGTAGAGAAATACGCGACAACATCAAATC	65
Pool3_mPox_AUMC_71_LEFT	TCATGCTGTAATCATATCCCCACTGA	66
Pool3_mPox_AUMC_71_RIGHT	GTTATATAGTGTAACATGAATGCAGTTTGGG	64
Pool3_mPox_AUMC_74_LEFT	GAATATATAAGACACAGAAATACTATATCCGGCA	64
Pool3_mPox_AUMC_74_RIGHT	CGAGTACTGATTCCAAGTTGCCAG	66
Pool3_mPox_AUMC_82_LEFT	TCATGCCATTATGATAAGTACCCTTATATCCA	65
Pool3_mPox_AUMC_82_RIGHT	TTTTGACATTCTTTTAGCAATGATTCCACA	64
Pool3_mPox_AUMC_85_LEFT	AGAGTATCGTTGAGACTTCCCACAA	66
Pool3_mPox_AUMC_85_RIGHT	GCGTATCTTCATCCATTCTATAGCACAAATA	65
Pool3_mPox_AUMC_87_LEFT	TCAAGGATGTACAAGTATTAAAATCTACTTCCA	64
Pool3_mPox_AUMC_87_RIGHT	CATTAATATGTACATATTGCAAGTCATTCTCGG	64
Pool3_mPox_AUMC_91_LEFT	TCATGACAATTCTAACGAATATGTGGATAAAGA	64
Pool3_mPox_AUMC_91_RIGHT	CGAGGAGGATATTTATGTCTATCCAGTAGA	65
Pool3_mPox_AUMC_93_LEFT	ACATATCCAGATATCTATATTCCTACTAGTTTGCT	64
Pool3_mPox_AUMC_93_RIGHT	TTCAAGAAAGGTGGGTGGAGAGAA	66
Pool3_mPox_AUMC_96_LEFT	GAGTATCCGGATACACGTCTACCG	67
Pool3_mPox_AUMC_96_RIGHT	AGAGAGAAAGGATAAAAACTTTTTACGACTCC	65
Pool4_mPox_AUMC_12_LEFT	CAGTAATATTGTTGATTATTTCTGACATCGACG	64
Pool4_mPox_AUMC_12_RIGHT	GTTCATGAAGGAGTATCTTACGAAGTATTTGA	64
Pool4_mPox_AUMC_16_LEFT	CATTGTTTGTTCCCTGGAGTATGAGAG	66
Pool4_mPox_AUMC_16_RIGHT	CCTAGGTCTGGTCTGTCCCTAAAAG	67
Pool4_mPox_AUMC_20_LEFT	GGGACATTATGTTGTTAAAATATAAACAGTCTCC	64
Pool4_mPox_AUMC_20_RIGHT	TCTATAATAATACATTAATATCATCCGACGGTGC	64
Pool4_mPox_AUMC_22_LEFT	CATTCAAATGTTGTCAAATGATCGGATCT	64
Pool4_mPox_AUMC_22_RIGHT	CGGGAGACACGGATTTATTAATATCGAAA	65
Pool4_mPox_AUMC_26_LEFT	TGTACTAGCTCTCAAGTATCTAGTTAGCAA	64

Pool4_mPox_AUMC_26_RIGHT	AAGATGCAGGTAATAATACATCAGGTTAAAGT	64
Pool4_mPox_AUMC_30_LEFT	CTACAAGAATTTTGGAGAGCCTTAACGA	65
Pool4_mPox_AUMC_30_RIGHT	CCATTCTCAGATCTATAGAAGGTGAACTAGAAT	65
Pool4_mPox_AUMC_37_LEFT	ATGCATAACTACTCCTCCGTTGTTTTT	65
Pool4_mPox_AUMC_37_RIGHT	CTTCATACTTATCTGATATCTTTCCATCCTTCTT	64
Pool4_mPox_AUMC_4_LEFT	ACACAAATCATCTACGTTTATAACAACATTCTC	63
Pool4_mPox_AUMC_4_RIGHT	AATAGATCAATGACTACTATTCACTACAACGAC	64
Pool4_mPox_AUMC_41_LEFT	AATTGTTAGATTTTTCCGCAATAACATGGT	64
Pool4_mPox_AUMC_41_RIGHT	CCATCCTTTAAGAGACCCGGATAATTAATC	65
Pool4_mPox_AUMC_45_LEFT	AACTAACGATTACATCACCTCTAACATCATC	64
Pool4_mPox_AUMC_45_RIGHT	ATTTGTGTAAAGATCTATTATCATCCATCCGT	64
Pool4_mPox_AUMC_49_LEFT	ACATTATCTAGTTGTTTATGTGGTGCTACC	65
Pool4_mPox_AUMC_49_RIGHT	AGTATAGGATTCAGACATAATGTTTGACGAAA	64
Pool4_mPox_AUMC_54_LEFT	TGTCCATAATCCTCTACCATAGTAACACG	66
Pool4_mPox_AUMC_54_RIGHT	CTCTCCATCTAATCATCATATTCTTCTGTCG	64
Pool4_mPox_AUMC_56_LEFT	CTCTCATATTCAGCAGTTTATTGTCGTACT	64
Pool4_mPox_AUMC_56_RIGHT	AGTTATTGCCATTAATGCCATGTTTAATAGC	64
Pool4_mPox_AUMC_60_LEFT	ATTGTTGCCTCCTTTGACGGC	67
Pool4_mPox_AUMC_60_RIGHT	TCCTGAATATAATAGAGCTGTTAGGTTTGC	64
Pool4_mPox_AUMC_66_LEFT	GGGATCCATTGTTACCACGTCTGT	67
Pool4_mPox_AUMC_66_RIGHT	TGCCGCTCTAATTGTTCTGTTGGA	67
Pool4_mPox_AUMC_68_LEFT	CCCATGTTTCATCTATCATAGATTTAAACGC	64
Pool4_mPox_AUMC_68_RIGHT	CATGTTAAAAATGTCAGCTGCCGAC	65
Pool4_mPox_AUMC_72_LEFT	ATCCGAAATATTAAACCAGACAACCCC	65
Pool4_mPox_AUMC_72_RIGHT	GGTAACAACATTACCAATTTTTGAATCAATCG	64
Pool4_mPox_AUMC_8_LEFT	CATACTGTATGTTGCTACATGTAGGTATTCC	65
Pool4_mPox_AUMC_8_RIGHT	TTTATCATGAATGTGTATAATAAAGCCGATTCC	63
Pool4_mPox_AUMC_80_LEFT	ATATGTGTGCGCAATCAAAGTGGA	66
Pool4_mPox_AUMC_80_RIGHT	ATTCATAGTGGGTACAGTACATGTTGAATAAA	64
Pool4_mPox_AUMC_83_LEFT	AGATGTAGAATCAATGATATGGCACGC	65
Pool4_mPox_AUMC_83_RIGHT	GGCATCAACCATTTTGCTTTAAAGTATACG	65
Pool4_mPox_AUMC_90_LEFT	TGACGGGTGGTCCAAAACTAATGA	67
Pool4_mPox_AUMC_90_RIGHT	GGCAATGATCCGTCGCTATATTCC	66
Pool4_mPox_AUMC_94_LEFT	GATAATAAACAATGTATCAACTGATGACAATCTCC	64
Pool4_mPox_AUMC_94_RIGHT	ACATGAGAGAAACAATATATACGAGTATAATACGG	64
Pool5_Pool1_mPox_AUMC_5_LEFT	Pool5 consists of primers located in pool1-4.	
Pool5_Pool1_mPox_AUMC_5_RIGHT		
Pool5_Pool1_mPox_AUMC_9_LEFT		
Pool5_Pool1_mPox_AUMC_9_RIGHT		
Pool5_Pool1_mPox_AUMC_61_LEFT		
Pool5_Pool1_mPox_AUMC_61_RIGHT		
Pool5_Pool1_mPox_AUMC_78_LEFT		
Pool5_Pool1_mPox_AUMC_78_RIGHT		
Pool5_Pool3_mPox_AUMC_82_LEFT		
Pool5_Pool3_mPox_AUMC_82_RIGHT		

Pool5_Pool2_mPox_AUMC_84_LEFT
Pool5_Pool2_mPox_AUMC_84_RIGHT
Pool5_Pool3_mPox_AUMC_87_LEFT
Pool5_Pool3_mPox_AUMC_87_RIGHT
Pool5_Pool1_mPox_AUMC_92_LEFT
Pool5_Pool1_mPox_AUMC_92_RIGHT
